# Lactation and breast cancer risk: a case–control study in Connecticut

**DOI:** 10.1054/bjoc.2001.1793

**Published:** 2001-06

**Authors:** T Zheng, T R Holford, S T Mayne, P H Owens, Y Zhang, B Zhang, P Boyle, S H Zahm

**Affiliations:** Department of Epidemiology and Public Health, Yale University School of Medicine, 60 College, New Haven, CT 06520, USA; Department of Epidemiology and Biostatistics, McGill University, Montreal, Canada, H3A1A2; Department of Epidemiology and Biostatistics, European Institute of Oncology, Milan, Italy, 20141; Division of Epidemiology and Genetics, National Cancer Institute, Bethesda, 20892 USA

**Keywords:** breast cancer, breastfeeding, lactation, case–control study

## Abstract

In this report, we examined the relationship between lactation and breast cancer risk, in a case–control study of breast cancer, conducted in Connecticut between 1994 and 1998. Included were 608 incident breast cancer cases and 609 age frequency matched controls, aged 30–80 years old. Cases and controls were interviewed by trained study interviewers, using a standardized, structured questionnaire, to obtain information on lactation and other major risk factors. Parous women who reported ever lactation had a borderline significantly reduced risk of breast cancer (OR = 0.83, 95% CI, 0.63–1.09). An OR of 0.53 (95% CI, 0.27–1.04) was observed in those having breastfed more than 3 children compared to those who never lactated. Women having breastfed their first child for more than 13 months had an OR of 0.47 (95% CI, 0.23–0.94) compared to those who never breastfed. Lifetime duration of lactation also showed a risk reduction while none of the ORs were statistically significant. Further stratification by menopausal status showed a risk reduction related to lactation for both pre- and postmenopausal women, while the relationship is less consistent for the latter. These results support an inverse association between breastfeeding and breast cancer risk. © 2001 Cancer Research Campaign http://www.bjcancer.com


*British Journal of Cancer* (2001) **84**(11), 1472–1476
© 2001 Cancer Research Campaign
doi: 10.1054/ bjoc.2001.1793, available online at http://www.idealibrary.com on IDEAL®

http://www.bjcancer.com

### Lactation and breast cancer risk: a case–control study in Connecticut

**T Zheng[1], TR Holford[1], ST Mayne[1], PH Owens[1], Y Zhang[1], B Zhang[2], P Boyle[3] and SH Zahm[4]**

[1]Department of Epidemiology and Public Health, Yale University School of Medicine, 60 College, New Haven, CT 06520, USA; [2]Department of Epidemiology and Biostatistics, McGill University, Montreal, Canada, H3A1A2; [3]Department of Epidemiology and Biostatistics, European Institute of Oncology, Milan, Italy 20141; [4]Division of Epidemiology and Genetics, National Cancer Institute, Bethesda, 20892 USA

**Summary** In this report, we examined the relationship between lactation and breast cancer risk, in a case–control study of breast cancer, conducted in Connecticut between 1994 and 1998. Included were 608 incident breast cancer cases and 609 age frequency matched controls, aged 30–80 years old. Cases and controls were interviewed by trained study interviewers, using a standardized, structured questionnaire, to obtain information on lactation and other major risk factors. Parous women who reported ever lactation had a borderline significantly reduced risk of breast cancer (OR = 0.83, 95% CI, 0.63–1.09). An OR of 0.53 (95% CI, 0.27–1.04) was observed in those having breastfed more than 3 children compared to those who never lactated. Women having breastfed their first child for more than 13 months had an OR of 0.47 (95% CI, 0.23–0.94) compared to those who never breastfed. Lifetime duration of lactation also showed a risk reduction while none of the ORs were statistically significant. Further stratification by menopausal status showed a risk reduction related to lactation for both pre- and postmenopausal women, while the relationship is less consistent for the latter. These results support an inverse association between breastfeeding and breast cancer risk. © 2001 Cancer Research Campaign http://www.bjcancer.com

**Keywords**: breast cancer; breastfeeding; lactation; case–control study

A number of epidemiological studies have investigated the relationship between breastfeeding and breast cancer risk. However, the association between lactation and breast cancer risk remains inconclusive. For example, some studies suggest that the inverse association may exist only among premenopausal women (Yang et al, 1993; Newcomb et al, 1994; Katsouyanni et al, 1996), particularly among those with a longer duration of breastfeeding (Freudenheim et al, 1997; Newcomb et al, 1999; Furberg et al, 1999) and with early age at first breastfeeding (Newcomb et al, 1999; Brinton et al, 1995). Some studies have found a protective effect from breastfeeding only among postmenopausal women (McTiernan and Thomas, 1986; Romieu et al, 1996; Freudenheim et al, 1997; Newcomb et al, 1999), which does not seem to vary with age at first or last lactation (Freudenheim et al, 1997; Furberg et al, 1999; Zheng et al, 2000a). Several studies also show that it is the duration of breastfeeding for the first child, not the lifetime duration of breastfeeding, which may determine the inverse association between breastfeeding and breast cancer risk (Byers et al, 1985; Siskind et al, 1989; Romieu et al, 1996). The results relating number of children breastfed to breast cancer risk have also been inconsistent (Yoo et al, 1992; Romieu et al, 1996; Furberg et al, 1999; Newcomb et al, 1999).

It is of importance to clarify the relationship between breastfeeding and breast cancer risk, not only because breastfeeding is a modifiable factor, but also because understanding the role of breastfeeding may contribute to our knowledge about the aetiology of a disease with significant public health consequence (Freudenheim et al, 1997). In this report, we present the results related to breastfeeding and breast cancer risk, based on data from a case–control study of breast cancer conducted in Connecticut between 1994 and 1997.

*Received 15 November 2000*
*Revised 12 February 2001*
*Accepted 12 February 2001*

*Correspondence to:* T Zheng

### MATERIALS AND METHODS

### Study population

A description of the study population and methods has been given elsewhere (Zheng et al, 2000b). Briefly, cases for the case–control study were histologically confirmed, incident breast cancer patients (ICD-O, 174.0–174.9) who either had breast-related surgery at the Yale-New Haven Hospital (YNHH), in New Haven County, or who were residents of Tolland County, Connecticut, between January 1, 1994 and December 31, 1997. Subjects were restricted to women 30 to 80 years of age who had no previous diagnosis of cancer, with the exception of non-melanoma skin cancer, and who were alive at the time of interview.

In Tolland County, newly diagnosed cases were identified from area hospital records, by the Rapid Case Ascertainment Shared Resource of the Yale Comprehensive Cancer Center. A total of 238 Tolland County cases were identified for this study with 176 of them (74%) completing in-person interviews. Population-based controls were recruited using either random digit dialing methods for those below age 65 as described by Hartge and colleagues (Hartge et al, 1984) or, from Health Care Finance Administration files for those aged 65 and above. A total of 322 Tolland County controls were randomly selected, and 206 (64%) agreed to participate in this study. Efforts were made to frequency match the cases and controls by age (within 5-year intervals) using a 1:1 ratio by adjusting the number of controls randomly selected in each age stratum every few months.

We also recruited both cases and controls from New Haven county, identified using computerized patient information from YNHH, where records of all newly performed breast-related surgeries are kept. All breast cancer patients who met the study eligibility requirements as described above, were consecutively as cases. A total of 562 incident breast cancer cases were identified from YNHH, with 433 of them (77%) completing in-person interviews. From the same YNHH computerized files, we also randomly selected 571 potential control patients who had had breast-related surgery and who were histologically diagnosed with normal tissue or benign breast diseases. Of these, 406 (71%) participated in the study. Efforts were made to frequency match the YNHH cases and controls by age (within 5-year intervals) using a 1:1 ratio by adjusting the number of controls randomly selected in each age stratum every few months. Of the 406 controls, 47 were given a normal diagnosis, 60 were diagnosed with fibroadenomas, 122 with other nonproliferative benign breast diseases, and 177 with proliferative benign breast diseases without atypia. Subjects with atypical hyperplasia were excluded from the study.

Pathological information for all the breast cancer cases and for the YNHH controls was reviewed by a single reference pathologist for diagnostic confirmation and uniform histological confirmation. Carcinomas were classified as in situ, invasive ductal, or invasive lobular, and were staged according to the TNM system (Beahrs and Myers, 1983).

### Interviews

After approval by the hospitals and each subject's physician, or following selection through random sampling, potential participants were approached by letter and then by phone. Trained study interviewers interviewed those who agreed, either in the subject's home or at a convenient location. A standardized, structured questionnaire was used to obtain information on menstrual and reproductive factors. The respondents were asked whether they had ever been pregnant, and about how many pregnancies they had had, at what age they had their first live birth, and for each live birth, whether they breastfed their infants. If their response was affirmative, the respondents were then asked about how many months they breastfed each infant, and about how many months they breastfed each infant with breast milk only, using no other food or formula.

The same questionnaire was also used to collect information on other potential confounding factors, including family cancer history, occupation, past medical history, exposure to electromagnetic fields, and demographic factors. Dietary information was collected using a scanable semi-quantitative food frequency questionnaire developed by the Fred Hutchinson Cancer Research Center. Each subject was asked to characterize her usual diet in the year prior to being interviewed. The entire interview took about 60 to 90 minutes to complete. All procedures were performed in accordance with a protocol approved by the Yale Human Investigations Committee.

### Data analysis

For the purposes of this analysis, we excluded nulliparous women or women whose pregnancies did not yield live births (86 cases and 98 controls), since earlier studies suggest that a nulliparous status may carry higher risk of breast cancer, and inclusion of nulliparous women in the 'never lactated' reference category may result in a stronger or artificial inverse association (Furberg et al, 1999). To investigate the earlier observation that breastfeeding may affect pre- and postmenopausal women differently, data were stratified by menopausal status. We presented data by number of children breastfed, duration of breastfeeding the first child, and lifetime duration of breastfeeding. Since earlier studies suggest that age at lactation may impact the risk of breast cancer, we presented the data by age at first and at last lactation.

Unconditional logistic regression was used to estimate the association between various aspects of breastfeeding and breast cancer risk, and to control for potential confounders. Variables included in the final model include: age (as a continuous variable), body mass index (<21.0, 21.0–24.9, ≥25.0 kg/m$^2$), age at first full-term pregnancy (<20, 20–25, ≥26 years), number of live births (1, 2, ≥3), dietary fat intake in grams day$^{-1}$ (<46, 47–71, ≥72), income 10 years before disease diagnosis or interview (<$20 000, $20 000–24 999, ≥$25 000), race (whites, blacks/others), education (1–12, 13–15, ≥16 years); family breast cancer history, and study site (YNHH and Tolland County). We kept these major risk factors in the final model while most of them, except age, did not bring a material change to the risk estimates (the usual 10% rule), because some of the earlier studies have been criticised for not being able to adjust for these potential confounders, and we want to be certain that the known risk factors included in our analyses did not confound the estimates. Odds ratios (OR) and 95% confidence intervals were calculated using SAS statistical software (SAS Institute, 1990). Tests for trend were conducted by using a likelihood ratio statistic in a logistic regression model.

### RESULTS

The distribution of the selected characteristics of breast cancer cases and controls is presented in Table 1. Compared with the controls, cases were slightly older despite an attempt at matching, therefore, age was controlled for in all analyses. Cases also had higher body mass index, later age at first full-term pregnancy and higher dietary fat intakes. A positive family history of breast cancer was associated with a borderline significantly increased risk of breast cancer. These observations are generally in line with what is known about breast cancer aetiology.

Breastfeeding was reported by 42% of the breast cancer cases and 48% of the controls, which gave a covariate-adjusted OR of 0.83 (95% CI, 0.63–1.09, Table 2). Further stratification by menopausal status showed a nonsignificant 28% (OR = 0.73, 95% CI, 0.40–1.31) risk reduction for those reporting ever breastfeeding among premenopausal women. Among postmenopausal women, the corresponding covariate-adjusted OR was 0.91 (95% CI, 0.66–1.26).

The number of children breastfed showed a generally inverse association with breast cancer risk. An OR of 0.53 (95% CI, 0.27–1.04) was observed among those reporting to have breastfed more than 3 children when compared with those who never had breastfed. The corresponding OR was 0.43 (95% CI, 0.07–2.57) for premenopausal women and 0.56 (95% CI, 0.27–1.18) for postmenopausal women.

Duration of breastfeeding for the first live birth was significantly inversely associated with breast cancer risk. For women who reported breastfeeding their first child for more than 13 months, the OR was 0.47 (95% CI, 0.23–0.94) when compared to those who never breastfed. Stratification by menopausal status

© 2001 Cancer Research Campaign

**Table 1** Selected characteristics of breast cancer cases and controls in Connecticut

| Characteristics | Cases (*n* = 522) | Controls (*n* = 511) | OR[a] | 95% CI |
|---|---|---|---|---|
| Age (years) | | | | |
| <50 | 147 | 184 | 1.00 | |
| ≥50 | 375 | 327 | 1.51 | 1.13–2.03 |
| BMI (kg/m$^2$) | | | | |
| <21 | 64 | 84 | 1.00 | |
| 21–24 | 208 | 192 | 1.52 | 1.02–2.24 |
| ≥25 | 250 | 235 | 1.34 | 0.90–1.98 |
| Age at menarche (years) | | | | |
| ≥15 | 59 | 55 | 1.00 | |
| 13–14 | 210 | 221 | 0.85 | 0.56–1.31 |
| <13 | 248 | 234 | 0.91 | 0.60–1.39 |
| Unknown | 5 | 1 | – | |
| Age at first full-term pregnancy (years) | | | | |
| <20 | 53 | 73 | 1.00 | |
| 20–25 | 260 | 240 | 1.54 | 1.01–2.34 |
| ≥26 | 209 | 197 | 1.57 | 1.00–2.48 |
| Unknown | 0 | 1 | – | |
| Number of live births | | | | |
| 1 | 86 | 87 | 1.00 | |
| 2 | 214 | 189 | 1.21 | 0.83–1.75 |
| ≥3 | 222 | 235 | 0.88 | 0.60–1.30 |
| Fat intake (g day$^{-1}$) | | | | |
| <46 | 143 | 172 | 1.00 | |
| 46–71 | 199 | 171 | 1.45 | 1.07–1.99 |
| ≥72 | 165 | 150 | 1.43 | 1.04–1.98 |
| Unknown | 15 | 18 | 1.12 | 0.54–2.35 |
| Annual income ($) | | | | |
| <20 000 | 318 | 308 | 1.00 | |
| 20 000–24 999 | 38 | 52 | 0.62 | 0.39–0.98 |
| ≥25 000 | 71 | 75 | 0.88 | 0.60–1.28 |
| Unknown | 95 | 76 | 1.11 | 0.77–1.58 |
| Family breast cancer history | | | | |
| No | 399 | 405 | 1.00 | |
| Yes | 123 | 106 | 1.24 | 0.91–1.68 |
| Race | | | | |
| Whites | 473 | 468 | 1.00 | |
| Others | 49 | 43 | 0.77 | 0.49–1.23 |

[a]Odds ratios for each selected characteristic were adjusted for all other selected characteristics listed in Table 1.

also showed a risk reduction among both premenopausal (OR = 0.53, 95% CI, 0.18–1.56) and postmenopausal women (OR = 0.46, 95% CI, 0.17–1.25) although the association was no longer statistically significant due to a diminished sample size after stratification.

Lifetime duration of breastfeeding also showed a risk reduction, particularly among premenopausal women. However, none of the ORs were statistically significant. For women who reported a lifetime history of breastfeeding for more than 13 months, the OR was 0.74 (95% CI, 0.36–1.52) for premenopausal women and 0.88 (95% CI 0.54–1.41) for postmenopausal women when compared to those who never breastfed.

Age at first or last period of lactation was not consistently associated with breast cancer risk (Table 3). Among postmenopausal women, a 43% (OR = 0.57, 95% CI, 0.25–1.29) nonsignificant risk reduction was observed for those who reported the first period of breastfeeding as occurring before age 25. Among postmenopausal women, however, a nonsignificant 55% (OR = 0.45, 95% CI, 0.17–1.19) risk reduction was observed for those who reported the first period of breastfeeding as occurring at age 35 and over. There is no clear risk pattern associated with age at last lactation.

### DISCUSSION

The results from this study suggest an inverse association between breastfeeding and breast cancer risk. The relationship, however, seems more consistent among premenopausal women, and stronger among those who breastfed more than 3 children and whose duration of breastfeeding was longer, particularly with regard to breastfeeding their first child.

Several mechanisms have been proposed to support a reduced risk of breast cancer associated with prolonged lactation (Freudenheim et al, 1997; Furberg et al, 1999; Newcomb et al, 1999), these include (1) a reduced exposure to the cyclic hormones of reproductive life due to ovulatory suppression occurring with prolonged breastfeeding; (2) a protective effect from direct physical changes in the breast that accompany milk production; (3) a reduction in the concentrations of toxic organochlorines in the breast with increasing cumulative duration of lactation; and (4) an expression of transforming growth factor-β during lactation, a hormonally regulated negative growth factor in human breast cancer cells.

As discussed earlier, however, the relationship between breastfeeding and breast cancer risk according to menopausal status has

 © 2001 Cancer Research Campaign

**Table 2** Breast cancer risk associated with lactation among parous women in Connecticut

| Variable | All subjects | | | Premenopausal | Postmenopausal |
|---|---|---|---|---|---|
| | Cases | Controls | OR[†] (95% CI) | OR[a] (95% CI) | OR[a] (95% CI) |
| Never | 301 | 267 | 1.00 | 1.00 | 1.00 |
| Ever | 221 | 244 | 0.83 (0.63–1.09) | 0.73 (0.40–1.31) | 0.91 (0.66–1.26) |
| Number of children breastfed | | | | | |
| 0 | 301 | 267 | 1.00 | 1.00 | 1.00 |
| 1 | 90 | 96 | 0.85 (0.60–1.21) | 0.63 (0.29–1.34) | 1.03 (0.68–1.58) |
| 2 | 79 | 90 | 0.76 (0.51–1.13) | 0.77 (0.37–1.60) | 0.74 (0.45–1.24) |
| 3 | 36 | 30 | 1.30 (0.74–2.28) | 0.73 (0.21–2.55) | 1.70 (0.86–3.38) |
| >3 | 16 | 28 | 0.53 (0.27–1.04) | 0.43 (0.07–2.57) | 0.56 (0.27–1.18) |
| *P* for trend | | | 0.10 | 0.25 | 0.27 |
| Duration of breastfeeding first child (in months) | | | | | |
| 0 | 329 | 300 | 1.00 | 1.00 | 1.00 |
| 1–6 | 134 | 136 | 0.90 (0.66–1.22) | 0.77 (0.41–1.47) | 0.95 (0.66–1.37) |
| 7–12 | 45 | 48 | 0.79 (0.49–1.26) | 0.49 (0.21–1.17) | 1.15 (0.62–2.12) |
| 13 | 14 | 27 | 0.47 (0.23–0.94) | 0.53 (0.18–1.56) | 0.46 (0.17–1.25) |
| *P* for trend | | | 0.04 | 0.09 | 0.47 |
| Duration of lifetime breastfeeding (in months) | | | | | |
| 0 | 301 | 267 | 1.00 | 1.00 | 1.00 |
| 1–6 | 96 | 100 | 0.86 (0.61–1.21) | 0.77 (0.36–1.63) | 0.89 (0.60–1.33) |
| 7–12 | 46 | 50 | 0.82 (0.52–1.29) | 0.69 (0.30–1.60) | 1.03 (0.57–1.85) |
| 13 | 79 | 94 | 0.78 (0.53–1.14) | 0.74 (0.36–1.52) | 0.88 (0.54–1.41) |
| *P* for trend | | | 0.16 | 0.39 | 0.61 |

[a]Adjusted for age, body mass index (<21.0, 21.0–24.9, ≥25.0 kg/m$^2$), age at first full-term pregnancy (<20, 20–25, ≥26 years), number of live births (1, 2, ≥3), dietary fat intake in grams/day (<46, 47–71, ≥72), income 10 years before disease diagnosis or interview (<$20 000, $20 000–24 999, ≥$25 000), race (whites, blacks/others), education (1–12, 13–15, ≥16 years), family breast cancer history, and study site (YNHH, and Tolland County).

**Table 3** Risk of breast cancer associated with age at lactation in Connecticut women

| Variable | All subjects | | | Premenopausal | Postmenopausal |
|---|---|---|---|---|---|
| | Cases | Controls | OR[a] (95% CI) | OR[a] (95% CI) | OR[a] (95% CI) |
| Never | 301 | 267 | 1.00 | 1.00 | 1.00 |
| Age at first lactation[b] | | | | | |
| <25 | 92 | 109 | 0.77 (0.55–1.08) | 0.57 (0.25–1.29) | 0.84 (0.57–1.24) |
| 25–29 | 81 | 73 | 1.11 (0.76–1.65) | 0.94 (0.45–1.98) | 1.31 (0.79–2.15) |
| 30–34 | 33 | 39 | 0.84 (0.49–1.42) | 0.77 (0.33–1.78) | 1.02 (0.47–2.20) |
| ≥35 | 15 | 23 | 0.54 (0.27–1.09) | 0.72 (0.23–2.30) | 0.45 (0.17–1.19) |
| Age at last lactation | | | | | |
| <25 | 51 | 48 | 0.90 (0.57–1.42) | 0.84 (0.26–2.75) | 0.96 (0.58–1.59) |
| 25–29 | 65 | 70 | 0.85 (0.57–1.26) | 0.68 (0.30–1.53) | 0.94 (0.58–1.52) |
| 30–34 | 63 | 79 | 0.76 (0.50–1.15) | 0.70 (0.33–1.45) | 0.95 (0.55–1.64) |
| ≥35 | 42 | 47 | 0.77 (0.47–1.25) | 0.85 (0.34–2.11) | 0.72 (0.38–1.36) |

[a]Adjusted for age, body mass index (<21.0, 21.0–24.9, ≥25.0 kg/m$^2$), age at first full-term pregnancy (<20, 20–25, ≥26 years), number of live births (1, 2, ≥3), dietary fat intake in grams/day (<46, 47–71, ≥72), income 10 years before disease diagnosis or interview (<$20 000, $20 000–24 999, ≥$25 000), race (whites, blacks/others), education (1–12, 13–15, ≥16 years), family breast cancer history, and study site (YNHH, and Tolland County). [b]Without adjusting for age at first full-term pregnancy.

been inconsistent in the literature. It is unclear how one might explain the contradictory findings. Stuver et al (1997) have suggested that recall bias between pre- and postmenopausal women may account for those studies that did not observe an inverse association among postmenopausal women. They have pointed out that the possible misclassification of breastfeeding history is more likely for the older postmenopausal women than for the younger premenopausal women. While the misclassification of breastfeeding history may not be differential by case–control status, the greater degree of nondifferential misclassification of breastfeeding history for the older women may obscure a relatively weak inverse association existing in postmenopausal women.

A major reason for the discrepant findings of earlier studies may be related to the generally shorter duration of lactation in Western populations. A protective effect associated with a longer duration of breastfeeding has been quite consistently reported in countries where the prevalence of prolonged breastfeeding is high (Tao et al, 1988; Yuan et al, 1988; Wang et al, 1992; Yoo et al, 1992; Hirose et al, 1995; Romieu et al, 1996; Zheng et al, 2000a). For example, 4 studies from China (Tao et al, 1988; Yuan et al, 1988; Wang et al, 1992; Zheng et al., 2000a), where more than half the women lactate for at least 3 years, suggest that long-term lactation is protective among both pre-and postmenopausal women. It will be very interesting to see if the risk will change for the new generations in China who tend to marry later, and who have been

© 2001 Cancer Research Campaign 

subjected to the one-child policies implemented in the past decade, particularly in the large cities.

In interpreting the results from our current study, several potential limitations need to be considered. A potential concern is the study sample size that is relatively small, particularly after stratification by menopausal status. Another potential concern is the use of benign breast disease patients as part of the control group, and that the risk of benign breast disease may be inversely associated with breastfeeding. This seems unlikely since the observed proportion of subjects breastfeeding in this population is remarkably similar to that reported by other study populations (Furberg et al, 1999).

In summary, an inverse association between breastfeeding and breast cancer risk was found in this study among both pre- and postmenopausal women. It should be noted, however, that despite the tremendous effort that has been made, the relationship between various aspects of breastfeeding and subsequent breast cancer risk continues to be as controversial as ever. Considering the fact that breastfeeding is one of the few potentially modifiable factors in preventing breast cancer, the suggested protective effect from breastfeeding and the proposed carcinogenic mechanisms merit further investigation.

### ACKNOWLEDGEMENTS

This study is supported by a grant #CA-62986 from National Cancer Institute/National Institute of Environmental Health Science. The authors thank the study interviewers, Donna Carrano, Melita Bosnyak, Heather Hutson and Sylvia Ullman for their high-quality interviewing. We also appreciate the support of personnel at the hospitals of Connecticut.

### REFERENCES

Beahrs OH and Myers MH (ed) (1983) *Manual for staging cancer, 2nd*. America Joint Committee on Cancer, pp 127–134, Philadelphia, PA: JB Lippincott

Brinton LA, Potischman NA, Swanson CA et al (1995) Breast-feeding and breast cancer risk. *Cancer Causes Control* **6**: 199–208

Byers T, Graham S, Rzepka T et al (1985) Lactation and breast cancer: evidence for a negative association in premenopausal women. *Am J Epidemiol* **121**: 664–674

Freudenheim JL, Marshall JR, Vena JE et al (1997) Lactation history and breast cancer risk. *Am J Epidemiol* **146**: 932–938

Furberg H, Newman B, Moorman P and Millikan R (1999) Lactation and breast cancer risk. *Int J Epidemiol* **28**: 396–402

Hartge P, Brinton LA, Rosenthal JF (1984) Random digit dialing selecting a population-based control group. *Am J Epidemiol* **120**: 825–833

Hirose K, Tajima K, Hamajima N, Inoue M, Takezaki T, Kuroishi T, Yoshida M and Tokudome S (1995) A large-scale, hospital-based case-control study of risk factors of breast cancer according to menopausal status. *Jpn J Cancer Res* **86**: 146–154

Katsouyanni K, Lipworth L, Trichopoulou A et al (1996) A case-control study of lactation and cancer of the breast. *Br J Cancer* **73**: 814–818

McTiernan A and Thomas DB (1986) Evidence for a protective effect of lactation on risk of breast cancer in young women: results from a case-control study. *Am J Epidemiol* **124**: 353–358

Newcomb PA, Storer BE, Longnecker MP et al (1994) Lactation and a reduced risk of premenopausal breast cancer. *N Engl J Med* **330**: 81–87

Newcomb PA, Egan KM, Titus-Ernstoff L et al (1999) Lactation in relation to postmenopausal breast cancer. *Am J Epidemiol* **150**: 174–182

Petrakis NL, Wrensch MR, Ernster VL et al (1987) Influence of pregnancy and lactation on serum and breast fluid estrogen levels: implications for breast cancer risk. *Int J Cancer* **40**: 587–591

Romieu I, Hernandez-Avila M, Lazcano E et al (1996) Breast cancer and lactation history in Mexican women. *Am J Epidemiol* **143**: 543–552

SAS Institute (1990) SAS/STAT user's guide. Version 6. Cary, NC:SAS Institute

Siskind V, Schofield F, Rice D and Bain C (1989) Breast cancer and breastfeeding: results from an Australian case-control study. *Am J Epidemiol* **130**: 229–236

Stuver SO, Hsieh CC, Butone E and Trichopoulos D (1997) The association between lactation and breast cancer in an international case-control study: a re-analysis by menopausal status. *Int J Cancer* **71**: 166–169

Tao SC, Yu MC, Ross RK et al (1988) Risk factors for breast cancer in Chinese women of Beijing. *Int J Cancer* **42**: 495–498

Wang, QS, Ross RK, Yu MC et al (1992) A case-control study of breast cancer in Tianjin, China. *Cancer Epidemiol Biomarkers Prev* **1**: 435–439

Yang CP, Weiss NS, Brand PR et al (1993) History of lactation and breast cancer risk. *Am J Epidemiol* **138**: 1050–1056

Yoo KY, Tajimi K, Kuroishi T et al (1992) Independent protective effect of lactation against breast cancer: case-control study in Japan. *Am J Epidemiol* **135**: 726–733

Yuan JM, Yu MC, Ross RK et al (1988) Risk factors for breast cancer in Chinese women in Shanghai. *Cancer Res* **48**: 1949–1953

Zheng T, Li D, Liu Y, Zhang B, Wang Y, Chen Y, Zhang Y and Ownes PH (2000a) Lactation reduces breast cancer risk in Shandong Province, China. *Am J Epidemiol* **152**: 1129–1135

Zheng T, Holford TR, Mayne ST et al (2000b) Exposure to electromagnetic fields from use of electric blankets and other in-home electrical appliances and breast cancer risk. *Am J Epidemiol* **151**: 1103–1111

*© 2001 Cancer Research Campaign*
